# Effects of selenium yeast and jujube powder dietary supplements on conformational and functional properties of post-mortem chicken myofibrillar protein

**DOI:** 10.3389/fnut.2022.954397

**Published:** 2022-08-08

**Authors:** Zhuo Wang, Chao Yang, Defu Tang, Xue Yang, Li Zhang, Qunli Yu

**Affiliations:** ^1^College of Food Science and Engineering, Gansu Agricultural University, Lanzhou, China; ^2^College of Animal Science and Technology, Gansu Agricultural University, Lanzhou, China

**Keywords:** selenium yeast, myofibrillar protein, conformation, functional properties, gel properties

## Abstract

The aim of the study was to evaluate the effects of selenium yeast and jujube powder on the structure and functional properties of post-mortem myofibrillar protein (MP) in white feather broilers. Changes in the structure (surface hydrophobicity, secondary structure, and tertiary structure), functional properties (solubility, turbidity, emulsifying, and foaming characteristics), and gel properties (gel strength, springiness, and water-holding capacity) of the MPs of white feather broiler, which were fed with different concentrations of selenium yeast or/and jujube powder (selenium yeast: 0,0.3, and 0.6 mg/kg; jujube powder: 8% to replace corn) for 42 days, were determined at 0, 24, and 72 h post-mortem. The results showed that with increasing concentrations of selenium yeast and jujube powder in the diet, the α-helix content, solubility, emulsification, and foaming of post-mortem chicken MP increased significantly (*P* < 0.05). The gel strength, springiness, and water-holding capacity of MP also increased, but the differences between the treatment groups were not significant (*P* > 0.05). In addition, the β-folding content and turbidity of MP decreased significantly (*P* < 0.05). Both the increase in selenium yeast levels and the addition of jujube powder improved the structural integrity and functional properties of MP. The best improvement effect was found in the combination group of high-dose selenium yeast and jujube powder, and there were significant interactions between them in the indices of α-helix, β-folding, turbidity, emulsification, and foam stability of MP. In conclusion, supplementing diets with seleniumyeast and jujube powder could maintain the structural stability of MPs in post-mortem chicken breast, leading to good functional properties. The results of this study may provide new insights into the effects of pre-slaughter feeding on post-mortem muscle MP conformation control and quality improvement.

## Introduction

Consumer awareness of the safety and nutritional attributes of meat products is expanding. In general, some native proteins have poor functional qualities that directly impact the ability of meat to meet and satisfy the different demands of the food business (1). The function and gelation properties of myofibrillar protein (MP), an important component of meat protein, are known to affect the sensory quality and yield of the final product (2–4). However, changes in protein molecular structures are a significant determinant of their functional qualities, and protein spatial conformations are tightly linked to their functions (5). Structure–function connection is critical in determining the viability and practicality of proteins as multifunctional ingredients in food. To meet consumer needs, the food industry has intensified its focus on using numerous methods to modify the molecular structure and functional performance of proteins and to construct novel meat products with desirable and enhanced functional qualities (6).

**FIGURE 1:**
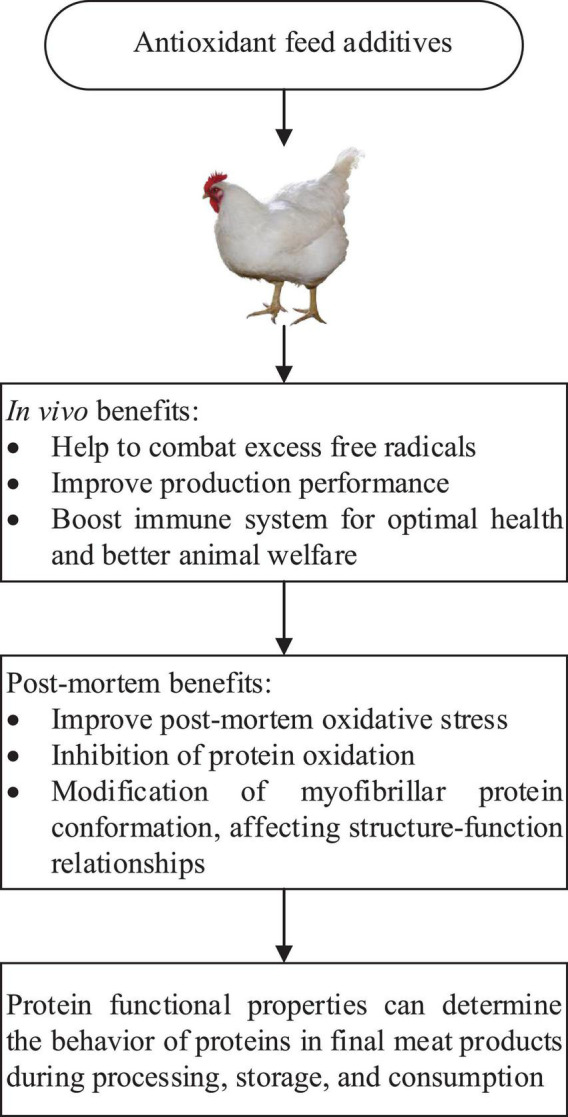
Schematic concept of post-mortem benefits of antioxidants in livestock.

Numerous modification methods, namely, additives (such as polysaccharides and polyphenols), oxidation (such as metal-catalyzed oxidation, lipid-oxidizing systems, and myoglobin-mediated oxidation), and new technologies (such as high hydrostatic pressure, electron beam irradiation, cold atmospheric plasma, and pulsed electric fields) (7–15), have been investigated to alter the functional properties of MP. However, the aforementioned innovative technologies often require high-level standard equipment and significant economic/energy input, resulting in a relatively high cost (16). Therefore, green processes based on the principle of green chemicals and clean processes that are friendly to resources and the environment are attractive for improving the functional quality of MPs. But how to combine additives and oxidation then affect in the modification of MPs and its properties, recent advances in antioxidant research have enabled meat scientists to think about the possibility of it in animal products through different feed strategies. For example, dietary glycerol monolaurate (GML) supplementation has the potential to modify the functional properties of egg white protein (EWP), and 450 mg/kg GML showed the most significant improvements in foaming capacity and foaming stability of EWP and the hardness of EWP gels (17). Borilova et al. (18) confirmed that selenium yeast feed supplements significantly improved the foaming capacity and foam stability of both albumen and yolk proteins. Dietary supplementation with 16% sea buckthorn pomace increased the total antioxidative capacity in lamb *longissimus thoracis* and improved the tenderness and water-holding capacity of the meat (19), and diet VE protected the catalytic sites of endogenous enzymes from being damaged by reactive oxygen species, thus increasing their hydrolytic activity and improving lamb tenderness (20). Li et al. (21) reported that organic selenium significantly increased the tenderness and solubility of chicken MPs by reducing their oxidation status. As shown in Figure 1, by reducing oxidative stress and oxidative rancidity in livestock and meat products, respectively, the biological effects of additional antioxidants may provide *in vivo* and post-mortem benefits. Xie et al. (22) demonstrated that adding up to 150 g/kg of dietary waste jujube to the meal improved goat body composition by increasing protein and essential amino acid (EAA) content and decreasing the ether extract (EE) content.

Antioxidants used in animal feeds are functionally considered to be similar to those used in food, and they can be broadly categorized into synthetic and natural. As an essential nutrient, selenium can improve the efficiency of the biological antioxidant system, thereby decreasing oxidative stress. Dietary selenium yeast supplementation can significantly increase the daily weight gain of broilers, improve GSH-Px, SOD, and T-AOC in the blood of laying hens, reduce drip loss, and stabilize meat color (23–25). In addition, natural antioxidants from various plants are rich in radical-scavenging polyphenols (26). The enzymatic breakdown of proteins (peptides) and cross-linking of small molecules into amphiphilic antioxidants suited for the interface have also been used to create “nature-origin” antioxidants (in emulsions, foams, etc.). Jujube, also referred to as Chinese date, is a sweet fruit with health benefits. Dried jujube has a high content of water-soluble carbohydrates, especially bioactive compounds, namely, polyphenols, flavonoids, organic acids, polysaccharides, and cyclic nucleosides (27–31), which can improve antioxidant capacity and regulate the immune response in animals (Table 1). Previous studies have indicated that waste jujube can be used as a substitute for wheat bran, barley, or corn in sheep diets (32, 33).

**TABLE 1:** Composition and nutrient levels of basal diets (as-fed basis).

Items	Diets					
	
	CK	CK+J	0.3S	0.6S	0.3S+J	0.6S+J
**Ingredients/%**						
Corn	58.77	50.77	58.77	58.77	50.77	50.77
Jujube meal		8.00			8.00	8.00
Soybean meal	35.62	35.62	35.62	35.62	35.62	35.62
Soybean oil	1.44	1.44	1.44	1.44	1.44	1.44
CaHPO_4_	1.74	1.74	1.74	1.74	1.74	1.74
Limestone meal	1.34	1.34	1.34	1.34	1.34	1.34
NaCl	0.20	0.20	0.20	0.20	0.20	0.20
Premix^a^	0.50	0.50	0.50	0.50	0.50	0.50
*DL*-Met	0.24	0.24	0.24	0.24	0.24	0.24
*L*-Lys⋅HCl	0.15	0.15	0.15	0.15	0.15	0.15
Total	100.00	100.00	100.00	100.00	100.00	100.00
**Extra added**						
Selenium yeast/(mg/kg)			0.30	0.60	0.30	0.60
**Nutrient levels**						
ME/(MJ/kg)^b^	14.20	14.34	14.47	14.58	14.72	14.97
CP	21.09	21.11	21.11	21.14	21.16	21.17
Ca	1.01	1.01	1.01	1.01	1.01	1.01
AP	0.48	0.48	0.48	0.48	0.48	0.48
Lys	1.21	1.21	1.21	1.21	1.21	1.21
Met	0.56	0.56	0.56	0.56	0.56	0.56

^a^The premix provided the following per kg of diets: VA 9 000 IU, VB_1_ 1.8 mg, VB_2_ 6.6 mg, VB_6_ 3 mg, VB_12_ 0.015 mg, VD_3_ 2000 IU, VE 27 IU, VK_3_ 2 mg, biotin 0.1 mg, folic acid 1.0 mg, nicotinic acid 30 mg, choline 250 mg, Cu (as copper sulfate) 10 mg, Fe (as ferrous sulfate) 50 mg, Mn (as manganese sulfate) 100 mg, Zn (as zinc sulfate) 66 mg, I (as potassium iodide) 1.0 mg.

^b^ME was a calculated value, while the others were measured values.

Most studies have been performed on the modification of the functional properties of egg white protein through feeding methods, and while published studies on feed additives have been related to the effects of additives on meat quality, little is known about the influence of feed additives on the functional properties of muscle proteins. The effect of Chinese jujube meal on conformational, functional, and gelling properties of MP of chicken carcass remains unclear. Hence, the aim of this study was to evaluate whether jujube can influence the structure of chicken MPs through feeding and change their functional and gelling properties.

## Materials and methods

### Animals and sample collection

A total of 360, 1-day-old white feather broilers (WOD168, China) with similar weights were randomly divided into six groups, with six replicates of 30 broilers in each group. The broilers were randomly divided into six groups according to the level of added jujube powder (jujube from Qinfengtan Jujube Garden, Minqin County, Wuwei City, and Gansu Province) and selenium yeast (purchased from a reagent company, trade name Lalmin Se2000, selenium content of 2 mg/g): CK (basic diet), CK+J (basic diet + 8% jujube powder instead of 8% maize), 0.3S (basic diet + 0.3 mg/kg selenium yeast), 0.6S (basic diet + 0.6 mg/kg selenium yeast), 0.3S+J (basic diet + 0.3 mg/kg selenium yeast + 8% jujube powder instead of 8% maize), and 0.6S+J (basic diet + 0.6 mg/kg selenium yeast + 8% jujube powder instead of 8% maize). The composition and nutritional levels of the basal diet are shown in Table 1, and the chemical composition and bioactive constituents of jujube powder are shown in Table 2. After 42 days of rearing in the same cage, at the same temperature, and in an *ad libitum* feeding and drinking water environment, chicken breast meat without visible fat was taken immediately after slaughter with reference to the GB/T 19478-2018 slaughter standard, and connective tissues were rapidly removed. All samples were matured for 0, 24, and 72 h post-mortem, then cut into 3 g pieces, packed into a holding tank at 4 ± 1°C, and brought back to the laboratory within 2 h for the determination of each index.

**TABLE 2:** Chemical composition and bioactive constituents of the jujube powder (DM basis).

Chemical composition	Contents (g/kg)	Bioactive constituents	Contents (g/kg)
Dry matter	815.3	Polysaccharides	68.72
Crude protein	67	Total amino acids	39.95
Neutral detergent fiber	126.3	Total EAA^a^	7.99
Acid detergent fiber	93.6	Methionine	0.26
Ether extract	35.2	Lysine	1.44
Ash	23.4	Threonine	1.25
Non-fiber carbohydrate	748.1	Valine	1.57
Starch	8.2	Isoleucine	0.96
Water-soluble sugar	730.9	Leucine	1.53
Glucose	21.95	Phenylalanine	0.88
Fructose	246.1	Proline	13.57
Rhamnose	131.2	Aspartic acid	10.64
Sucrose	20.5	Total triterpenoids	4198.51
Lignin	19	Total nucleosides	239.35
Ca	6.2	cAMP^b^	23.49
P	2.7	cGMP^c^	12.62

^a^Total EAA, total essential amino acids.

^b^cAMP, cyclic adenosine monophosphate.

^c^cGMP, cyclic monophosphate.

### Growth performance

All broilers in each replicate cage were weighed, respectively, after a 12-h fasting on 42 days of age to calculate the average body weight (ABW) of each treatment.

### Extraction of myofibrillar protein

MP was extracted from the chicken breast according to the method described by Cao et al. (34), stored in a tightly capped bottle at 4°C, and utilized within 24 h.

### Determination of the conformational properties of myofibrillar protein

#### Surface hydrophobicity

The actomyosin complex samples’ surface hydrophobicity was assessed using Chelh’s (35) methodology with a few changes. With 0.6 M KCl phosphate buffer (pH 7.5), the samples were diluted to 0.00125, 0.0025, 0.005, and 0.02 mg/ml, respectively. A total of 20 μL of 8 mM 8-anilino-1-naphthalenesulfonic acid (ANS) solution was then added to 4 ml of the sample solution. A SpectraMax microplate reader (Molecular Devices, Sunnyvale, CA) was used to detect the reaction solution at excitation wavelengths of 390 nm and 470 nm with a slit width of 3.0 nm. The slope of the relative fluorescence vs. the percent protein content (w/v) of the samples was used to determine their H_0_.

#### FT-IR spectra

By utilizing the technique described by Deng et al. (36), the Fourier Transform Infrared Spectroscopy (FT-IR) module (Tensor II, Bruker Co., United States) determined the infrared spectroscopy of MPs. Protein was produced by pressing protein isolates in a vacuum hydraulic press after mixing them with moisture-free potassium bromide at a ratio of 1:100 (w/w). FTIR spectra of protein isolate pellets was recorded in the range of 4000–400 cm^−1^.

#### Intrinsic fluorescence spectrophotometry

Fluorescence spectra were examined in order to learn more about the modification of protein tertiary structure. The experiment was carried out with a fluorescence spectrophotometer (F-7000, Hitachi Co., Tokyo, Japan) using a modified version of Xu et al.’s (37) approach. The freeze-dried sample was dissolved in 0.01 mol/L of phosphate buffer to create the sample solution (0.2 mg/ml) (pH 7.0). With a constant slit of 5 nm for both excitation and emission, the solution was excited at 290 nm, and emission spectra were obtained from 300 to 460 nm.

### Determination of the functional properties of myofibrillar protein

#### Solubility

The solubility of MPs, as defined by Agyare et al. (38), was calculated with minor adjustment. One gram of sarcoplasmic protein was removed from the muscle, inserting 20 ml of ice-cold 0.1 M phosphate buffer (pH 7.2, 1.1 M potassium iodide), shaking 20 s, 10,000 rpm for three times, and then left on a shaker at 4°C for 12 h. Samples were centrifuged at 1,500 *g* for 20 min, and the concentration of protein in the supernatants was measured by the Biuret process.


(1) $$S\,o\,l\,u\,b\,i\,l\,i\,t\,y\,\left(m\,g/g\right)=\frac{P\,r\,o\,t\,e\,i\,n\,c\,o\,n\,t\,e\,n\,t\,o\,f\,s\,u\,p\,e\,r\,n\,a\,t\,e}{T\,o\,t\,a\,l\,p\,r\,o\,t\,e\,i\,n\,c\,o\,n\,t\,e\,n\,t\,o\,f\,s\,a\,m\,p\,l\,e}$$

#### Turbidity

The turbidity was measured according to the method of Benjakul et al. (39), and 5 mL of protein solution which was adjusted to 1 mg/ml was added into a 10-ml centrifuge tube, heated in the water bath at 80°C for 30 min, and then cooled to room temperature. Protein aggregation was monitored continuously by measuring the absorbance at 660 nm.

#### Emulsifying activity and emulsifying stability

The emulsifying properties were measured according to Pearce and Kinsella (40) with slight changes. A total of 10 ml of corn oil (filter pressed corn oil, Yihai Kerry Arawana Co., Ltd., China) and 30 ml of 1 mg/ml of SMP solution in 0.1 M phosphate buffer (pH 7.0) were shaken together and then homogenized at 10,000 rpm for 1 min at room temperature. A total of 200 μL of emulsions were taken from the bottom of the container after being placed undisturbed for 10 min and dispersed into 10 ml of 0.1% SDS solution. The absorbance was recorded at 500 nm with 0.1% SDS as a blank control. Emulsifying activity index (EAI) and emulsion stability (ES) were defined as:


(2) $$E\,A\,I\,\left(m^{2}/g\right)=\frac{2\times 2.303}{C\times \left(1-\varphi \right)\times 10^{4}}\times A_{0}\times d\,i\,l\,u\,t\,i\,o\,n$$

(3) $$ES\;min=\frac{A_{10}}{A_{0}}\times 10$$

where A_0_ and A_10_ represent the absorption value of the emulsion at 0 and 10 min, respectively.

#### Foaming activity and foaming stability

Foaming properties were carried out by using the method of Agyare et al. (41). 50 mL of SMP solution (1 mg/mL, pH 7.0) foaming solution was add into a 100 mL graduated cylinder, and whipped with a rotating anchor at 15000 rpm for 2 min at room temperature, and the height V_0_ of foam was recorded after the end of whipping and V_30_ after 30 min. The foaming capacity (FC) and foaming stability (FS) were calculated according to the following formulas, respectively:


(4) $$F\,C\%=\frac{V_{0}-50}{100}\times 100\%$$

(5) $$F\,S\%=\frac{V_{30}-50}{V_{0}-50}\times 100\%$$

### Determination of the gelation properties of myofibrillar protein

#### Preparation of myofibrillar protein gelation

The MPs were suspended at a concentration of 40 mg/ml in phosphate buffer (20 mM, pH 7.0) containing 0.6 M NaCl. At a rate of 1°C min^–1^, the solutions were heated from 25 to 75°C. After that, the heat treatment was kept at 75°C for 20 min. The resultant samples were kept at 4°C for 12 h after being chilled to 4°C in an ice bath. The preparation of each gel sample was done in three copies.

#### Texture of myofibrillar proteins gelation

The method reported by Xia et al. (42) was somewhat modified to assess gel strength using a TA-XT texture analysis meter fitted with a cylindrical probe (P0.5 diameter). In a nutshell, each sample (15 mm × 15 mm × 15 mm) was subjected to two-cycle compression under the following circumstances: pretest speed of 5 mm/s^–1^, test and posttest speed of 1 mm/s^–1^, trigger force of 5 g, and ultimate distance of 30%. The breaking force (g) and distance (mm) were multiplied to determine the gel strength (g × mm).

#### Water-holding capacity of myofibrillar proteins gelation

The water-holding capacity (WHC) of the MP gels was calculated using a slightly modified version of the Xia et al. (42) approach. In a nutshell, the supernatant fluid was drained after centrifuging 3 g of the gel samples at 10,000 *g* for 15 min at 4°C. The weight of the gel after centrifugation to the weight of the gel before centrifugation was used to calculate WHC.

### Statistical analysis

SPSS software (SPSS, Version 20.0, IBM Co., Somers, NY, United States) was used for statistical analysis following the General Linear Models (GLM) procedure. The model included selenium yeast and jujube powder treatments (as fixed effects) and their interaction (S × J). A two-way analysis of variance (ANOVA) was used to analyze the interactions between fixed factors at a significance level of 0.05. After significant interaction (*P* < 0.05) between the storage and packaging treatment was noted on the variables, changes in conformational and functional properties among different post-mortem maturation for each treatment over various days were calculated by one-way analysis of variance. Duncan’s multiple range test was used to assess differences between mean values when *P* < 0.05.

## Results and discussion

### Growth performance

As shown in Table 3, compared with the CK group, the weight gain of broilers on day 42 in 0.6S and 0.6S+J groups was significantly increased (*P* < 0.05), by 6.07 and 6.12%, respectively. The average daily gain of 0.6S group and 0.6S +J group was significantly increased (*P* < 0.05), and increased by 6.24 and 6.28%, respectively. These results indicate that adding jujube powder and selenium yeast to the diet can significantly improve the growth performance of white-feathered broilers. Radmila et al. (23) also made a similar discovery by adding selenium to Lingnan yellow broilers. Selenium can improve the growth performance of broilers possibly because selenium is an important cofactor of 25’2 deiodinase, a key enzyme in the synthesis of Triiodothyronine (T3) in animals, and T3 is the main element in the regulation of growth and nutrition in animals, especially poultry, which can promote the assimilation of protein, thus significantly improving the weight gain of broilers. Dietary jujube powder can improve the growth performance of broilers, which may be related to the improvement of dietary palatability and increase in feed intake of broilers.

**TABLE 3:** Effect of dietary selenium yeast and jujube powder supplementation on growth performance of white feather broiler.

Items	Treatments						SEM	*P*
			
	CK	CK+J	0.3S	0.6S	0.3S+J	0.6S+J		
**ABW (g/bird)**								
Day 1	47.13	47.22	47.03	47.06	47.1	47.19	0.050	0.003
Day 42	1883.93	1960.527	1988.79	1983.36	1998.42	1999.17	1.948	0.418
ADG (g/bird/d) Day 1–42	43.73	45.56	46.23	45.86	46.46	46.48	0.956	0.333

ABW, average body weight; ADG, average daily gain.

### Changes in the conformational properties of myofibrillar protein

#### Surface hydrophobicity

**FIGURE 2:**
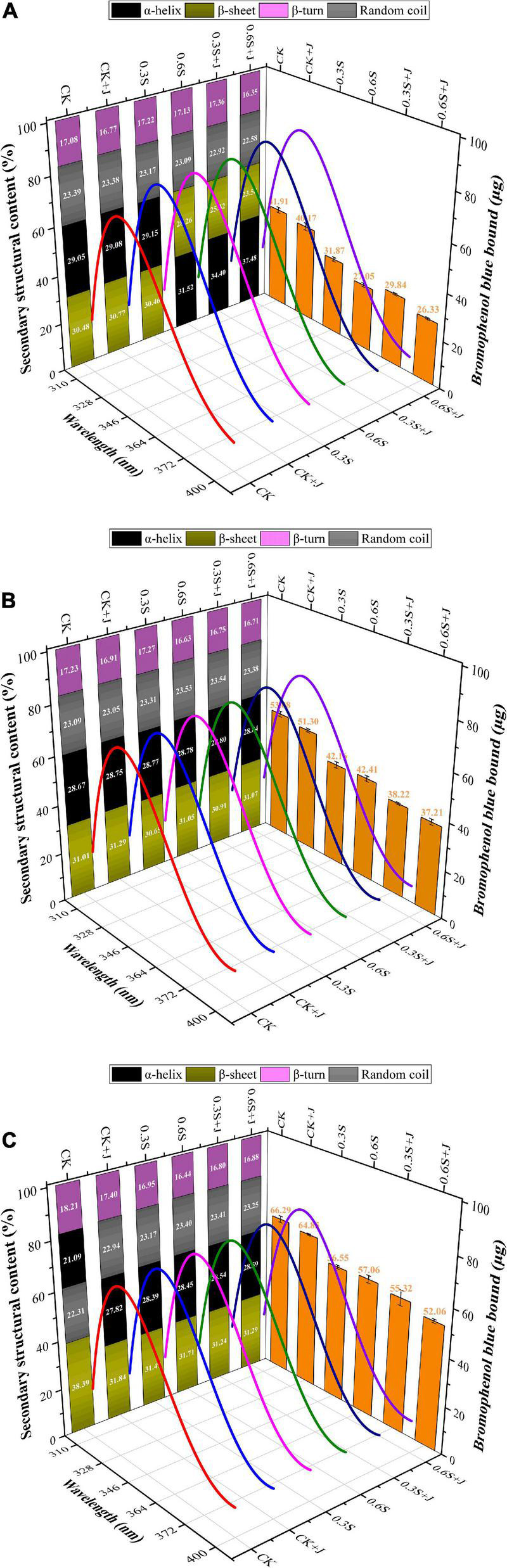
Changes in the secondary structural content (left side), surface hydrophobicity (right side), and intrinsic fluorescence spectroscopy (3D Space) of post-mortem chicken myofibrillar protein from selenium yeast and jujube powder dietary supplements. **(A)** Conformation changes at post-mortem 0 h, **(B)** conformation changes at post-mortem 24 h, and **(C)** conformation changes at post-mortem 72 h.

The primary mechanism preserving the protein’s tertiary structure is the interaction of hydrophobic groups. As a result, the distribution of hydrophobic groups on the protein surface can accurately represent the stability of the protein’s structure and its functional characteristics (43). As shown in Figure 2, surface hydrophobicity of all feeding treatments showed a significant increase with storage time (*P* < 0.05) and reached a maximum at 72 h among different feeding groups, which was consistent with the finding of Mignino et al. (44) on MP of the mantle from frozen-stored squid that surface hydrophobicity increased with aging time prolonged. More hydrophobic groups become exposed during storage, weakening the hydrophobic connection between the protein molecules and increasing the hydrophobicity of the protein’s surface (45). However, MP surface hydrophobicity at the same aging time was basically showing a downward trend with the supplement of selenium yeast and jujube powder. The resemblances among different post-mortem periods were that MP surface hydrophobicity of treatments fed with selenium yeast or jujube powder was significantly lower than that of the CK group, and the surface hydrophobicity of the treatments with jujube powder was lower than that of the treatments without jujube powder. The above results confirmed the significant interaction effect of selenium yeast and jujube powder (*P* < 0.05) and the significant main effect of selenium yeast or jujube powder (*P* < 0.01). Selenium is a component of a selenoprotein methionine sulfoxide reductase B, which prevents protein oxidation, a key factor in the deterioration of meat quality during storage (46). Because hydrophobic amino acids are buried within the protein, it is generally accepted that a decreased surface hydrophobicity suggests the folding state of the protein structure which depends on its oxidation degree. In addition, it is generally accepted that a decreased surface hydrophobicity suggests the folding state of the protein structure and that the hydrophobic amino acids are buried within the protein (47). Also, the non-covalent interactions that occur between polyphenols of jujube powder and proteins led to lots of hydroxyl groups linked at the aromatic ring being introduced to the surface of the protein molecule, thus decreasing the surface hydrophobicity (48, 49), which was consistent with the founding reported by Meng and Li (50) that the addition of rye polyphenols to the ration significantly reduced the surface hydrophobicity of loach.

#### FT-IR spectra

The α-helix and β-sheet characterize the regularity of protein molecules, which are stabilized by intra- and inter-hydrogen bonds between peptide chains (51), whereas random coils usually reflect a looser structure. It can be seen in Figure 2 that all treatments showed a similar changing trend, i.e., the content of α-helix gradually decreased, while that of β-sheet increased as maturation progressed. Similar to the study by Andersen et al. (52), extensive proteolysis of large structural proteins occurred post-mortem. Exposure of hydrophobic groups could lead to a reduction in intramolecular hydrogen bonds; therefore, the increase in β-sheet structure appeared to be due to the decrease in the α-helix and turn structure (53). Following the addition of selenium yeast and jujube powder to the diet, significant effects on the content of α-helix and β-sheet (*P* < 0.01) were observed, and there was a very significant interaction between these two additives. In the selenium yeast and jujube powder groups, there was an increase in α-helical content and a decrease in β-sheet content in chicken MP compared to that in the CK group. However, a significant difference was observed only between the high-dose selenium yeast and compound group, and no significant difference in α-helix and β-sheet content between the CK+J group and 0.3S group (*P* > 0.05) was found. There were no significant differences in the irregular curl content between the feeding groups. The highest α-helix content in chicken MP was in the 0.6 mg/kg selenium yeast and jujube powder group at 0 h post-mortem, which was 37.48%; meanwhile, the highest β-sheet content was found after 72 h of aging in the CK group, which was 38.39%, close to the level of highest α-helix content, which illustrated the switch between these two structures when the degree of protein oxidation was changed. Although there is little information regarding the effect of feed additives on protein secondary structure, existing research may provide evidence on how muscle antioxidant capacity is promoted by different dietary supplements. As an integral part of selenoproteins, dietary Se is a crucial factor regulating GSH-Px activity and the efficiency of the antioxidant system (54). By reducing the oxidation levels, the structural integrity of MP is maintained, leading to the prevention of damage to the hydrogen bonds within the α-helix and then turning into an unstable β-sheet (51).

#### Intrinsic fluorescence spectra

An essential metric for observing changes in the protein matrix is the intrinsic fluorescence spectrum. The primary fluorescent groups are aromatic residues, namely, tryptophan (Trp) and tyrosine (Tyr). The number of aromatic residues exposed inside the protein is represented by the relative intensity of the maximal fluorescence emission wavelength (λ_*max*_). The changes in the tertiary structure of MPs caused by feeding selenium yeast and jujube powder were evaluated by monitoring the changes in the inherent fluorescence intensity of Trp residues. The fluorescence emission spectra of MP modified by feeding selenium yeast and jujube powder compounds are shown in Figure 2. Considering the post-mortem effect, MP fluorescence decreased with prolonged aging within the same treatment group. At the same aging time, the MP of the CK group exhibited the lowest fluorescence intensity compared to that of all treatments. Trp residues are highly sensitive to the polarity of their microenvironment, suggesting that the protein fluorophore may have been altered as a result (55, 56). Trp residues are frequently found in the hydrophobic core of the folded proteins, where they display high quantum yield and high fluorescence intensity (57). Trp residues are exposed to a solvent (hydrophilic environment) when proteins become partially or entirely unfolded, which causes a decrease in fluorescence intensity. Therefore, the more complete the MP structure, the higher the observed fluorescence intensity. The 0.6 S + J group showed the highest fluorescence intensity, which is also shown in Figure 2 (left side). Selenium, an active component of GSH-Px, which is an endogenous antioxidant enzyme widely present in the animal body, participates in the defense system against animal peroxidation and catalyzes the oxidation of glutathione (GSH) to generate oxidized glutathione (GSSG), thus protecting the structure and function of cells (58). Generally, the λ_*max*_ of the intrinsic fluorescence of each treatment at the indicated post-mortem time points increases from 334 to 335 nm (Table 4). In contrast, the λ_*max*_ of intrinsic fluorescence at the same post-mortem time point decreased from 335 nm in CK to 334 nm in 0.6S+J. In other words, a red shift occurred during the post-mortem period, but a blue shift occurred with the addition of selenium yeast and jujube powder. This might be related to the stability of the protein structure, corresponding to an increase in the helix content in the secondary structure. Dietary selenium yeast and jujube powder may leave most of the secondary structural elements of MP intact by reducing its oxidation; therefore, as a sensitive index of conformational changes in the tertiary structure, Trp residues will fluoresce in a manner that is dependent on the folding of the protein, and the λ_*max*_ shift to shorter wavelengths (blue shift) indicating a decrease in the polarity of the Trp residue microenvironment. This might be attributed to the fact that antioxidant supplementation protects the internal hydrophobic interactions of protein molecules and induces molecular folding (36).

**TABLE 4:** λmax of intrinsic fluorescence spectroscopy of post-mortem chicken myofibrillar protein from selenium yeast and jujube powder dietary supplements.

λ max (nm)	CK	CK+J	0.3S	0.6S	0.3S+J	0.6S+J
0 h	334.00	335.00	334.00	334.00	334.00	334.00
24 h	335.00	335.00	335.00	334.00	334.00	334.00
72 h	335.00	335.00	335.00	335.00	335.00	335.00

### Changes in the functional properties of myofibrillar protein

#### Solubility

Solubility is a practical index to measure protein denaturation and aggregation, and it can affect many other functional properties of the protein. The solubility of MP decreased with the aging period (Table 5). Similar to the research of Xiong and Brekke (59) on protein denaturation in broiler breast meat, the extensive aggregation involving both disulfide bonds and non-disulfide interactions between protein subunits resulted in the solubility reduction which can be proved in the increase in surface hydrophobicity and a decline in α-helix. The addition of selenium yeast and jujube powder in the diet increased the solubility of chicken MP. At 0 h of the aging period, MP solubility of the highest-dose compound group was 34.84%, which was an increase of 33.79% compared with the CK group. However, except for the CK group, there was no significant difference in MP solubility in the other feeding groups; at the 24 and 72 h, the solubility of chicken MP also increased with the supplement of selenium yeast and jujube powder, but the differences were only significant between CK, CK+J, 0.3S, and other three treatments (*P* < 0.05). Studies have shown that adding selenium yeast to the diet can increase the solubility of chicken MP, but the difference is not significant (*P* > 0.05); the addition of jujube powder can significantly increase the solubility of chicken myofibril protein after slaughter (*P* > 0.05). Although the effect of the combination of the two is not significant (*P* > 0.05), the 0.6S+J feeding group has the best effect on increasing protein solubility. Xu et al. (7) ingested that high dietary valine decreased sarcoplasmic protein solubility, while it did not alter the solubility of MP and total protein relative to the ingestion of lower dietary valine (*P* > 0.05). Interestingly, Xu et al. (60) found that L-Val/H-Ile treatment led to the highest MP solubility and total protein solubility of pork. This demonstrated that dietary antioxidation could modify the solubility of animal protein, but depend on the kinds of muscle protein and the level of additions. Relkin and Shukat (61) found that the solubility of potato protein decreased due to polyphenols demonstrating that the precipitation was induced by protein–polyphenol conjugates rather than solubilization in an aqueous neutral media.

**TABLE 5:** Changes on functional properties of post-mortem chicken myofibrillar protein from selenium yeast and jujube powder dietary supplements.

Items	Time/h	Solubility/%	Turbidity/A_340_	EAI/m^2^/g	ES/%	FC/%	FS/%
CK	0	26.04 ± 3.15^b^	0.94 ± 0.07^a^	1.15 ± 0.09^Ca^	39.76 ± 4.88^Ca^	114.83 ± 1.89^c^	84.42 ± 1.41^Ac^
	24	21.35 ± 4.00^c^	0.97 ± 0.04^a^	1.46 ± 0.10^Ba^	54.61 ± 4.55^Ba^	112.50 ± 1.92^c^	78.15 ± 1.36^Bc^
	72	17.24 ± 3.54^b^	0.99 ± 0.03^a^	1.90 ± 0.17^Aa^	87.53 ± 7.76^Aa^	112.17 ± 2.79^c^	77.89 ± 1.40^Ca^
CK+J	0	29.76 ± 4.02^Bab^	0.81 ± 0.04^Bb^	0.88 ± 0.13^Cb^	37.07 ± 7.32^Cab^	119.25 ± 2.96^Ba^	87.20 ± 1.47^Aab^
	24	23.11 ± 3.04^Abc^	0.91 ± 0.04^Ab^	1.18 ± 0.14^Bb^	50.70 ± 6.61^Bab^	115.67 ± 2.79^Cb^	79.48 ± 1.32^Bbc^
	72	18.81 ± 3.88^Ab^	0.92 ± 0.03^Ab^	1.72 ± 0.26^Aab^	72.51 ± 10.24^Ab^	114.33 ± 1.89^Bbc^	77.48 ± 1.89^Ca^
0.3S	0	32.49 ± 3.54^Ca^	0.63 ± 0.04^Cc^	0.82 ± 0.11^Cbc^	33.75 ± 4.31^Cabc^	119.58 ± 2.06^Ab^	86.92 ± 1.52^Aab^
	24	26.04 ± 3.15^Bb^	0.76 ± 0.04^Bb^	1.17 ± 0.10^Bb^	47.24 ± 3.68^Bab^	118.92 ± 2.97^Aa^	79.78 ± 1.46^Bbc^
	72	18.81 ± 3.27^Ab^	0.86 ± 0.04^Ab^	1.66 ± 0.18^Ab^	71.80 ± 8.25^Ab^	114.33 ± 1.89^Bbc^	77.32 ± 1.30^Ca^
0.6S	0	32.88 ± 8.31^Ba^	0.53 ± 0.03^Bd^	0.75 ± 0.09^Bbc^	33.14 ± 3.90^Cbc^	119.50 ± 3.29^Ab^	86.77 ± 2.42^Abc^
	24	32.10 ± 5.91^Aa^	0.73 ± 0.12^Ab^	0.86 ± 0.09^Bc^	48.27 ± 6.62^Bab^	116.83 ± 2.09^ABab^	81.30 ± 2.05^Bab^
	72	24.87 ± 3.15^Aa^	0.79 ± 0.08^Abc^	1.62 ± 0.10^Ab^	64.12 ± 3.21^Abc^	115.00 ± 1.92^Bbc^	76.99 ± 1.29^Ca^
0.3S+J	0	33.27 ± 3.59^Ca^	0.47 ± 0.06^Ce^	0.74 ± 0.13^Bc^	27.78 ± 4.95^Ccd^	123.58 ± 2.06^Aa^	86.56 ± 2.08^Abc^
	24	31.71 ± 3.43^Ba^	0.68 ± 0.04^Bc^	0.85 ± 0.10^Bc^	48.34 ± 5.39^Bab^	119.00 ± 2.81^Ab^	82.05 ± 2.08^Ba^
	72	26.24 ± 4.29^Aa^	0.79 ± 0.04^Ad^	1.28 ± 0.12^Ac^	58.59 ± 4.47^Ac^	115.33 ± 1.89^Bbc^	76.67 ± 1.28^Ca^
0.6S+J	0	34.84 ± 3.54^Ba^	0.28 ± 0.03^Bf^	0.46 ± 0.09^Cd^	22.66 ± 4.71^Cd^	126.33 ± 2.14^Aa^	89.24 ± 1.52^Aa^
	24	32.49 ± 3.38^Ba^	0.36 ± 0.10^ABc^	0.64 ± 0.11^Bd^	45.98 ± 8.94^Bb^	119.25 ± 2.14^Ba^	83.33 ± 2.09^Ba^
	72	27.02 ± 4.16^Aa^	0.40 ± 0.04^Ae^	1.15 ± 0.15^Ac^	55.96 ± 6.18^Ac^	119.92 ± 2.97^Ba^	74.41 ± 1.87^Cb^
SEM		0.045	0.105	0.355	0.167	0.032	0.032
*P*	S	<0.01	<0.01	<0.01	<0.01	<0.01	<0.01
	J	<0.05	<0.01	<0.01	<0.05	<0.01	<0.01
	S × J	0.516	<0.01	0.861	0.985	0.434	0.434

Different lowercase letters represent the same time, and the difference between different treatment groups is significant (P < 0.05); different capital letters represent the difference between different time groups under the same diet (P < 0.05); in P-value, S represents the influence of Selenium yeast factor, J represents the influence of Jujube powder, and S × J represents the interaction effect of surface.

#### Turbidity

An essential metric for assessing protein binding and aggregation is turbidity. The degree of protein aggregation increases with increasing turbidity values (61). From Table 5, it can be seen that as storage time increased, the turbidity values increased for the control and addition supplementary groups (*P* < 0.05). This could be attributed to the increased degree of protein denaturation and aggregation, which affects light scattering and hinders light propagation, thus increasing the absorbance (62). With the increase of selenium yeast content in the diet, the turbidity is extremely reduced (*P* < 0.01); compared with the treatment group without jujube powder at the same level, the turbidity of the group treated with compound jujube powder was significantly lower (*P* < 0.01), and there was a significant interaction between selenium yeast and jujube powder (*P* < 0.05). Compared with the CK group, the MP turbidity in the group was reduced by 53–70%. Compared with the CK group, the addition of selenium yeast and jujube powder or the combination in the diet can significantly reduce the turbidity of chicken myofibril protein (*P* < 0.05). Studies have shown that both selenium yeast and jujube powder can reduce the turbidity level. Selenium yeast and jujube powder interact significantly (*P* < 0.05), and the combination of the two has the best effect. The 0.6S+J group chicken myofibril protein turbidity was the lowest among all treatment which indicated that the protein exhibits a higher degree of aggregation. Utrera et al. (63) studied the changes in beef quality during frozen storage and reported similar results that protein solubility represented contrast law to turbidity, the physical factors of antioxidants additives might protect the hydrogen bonds and hydrophobic interactions which were responsible for the intermolecular association of protein aggregates. For this reason, selenium yeast and jujube powder diet affected the oxidation degree of protein, hydrogen bonds inside the secondary structure were protected, and thus the hydrophobic groups were prevented from exposure, hence the solubility of MP increased and turbidity decreased.

#### Emulsifying activity and emulsifying stability

The emulsifying activity index (EAI) is determined by protein–protein and protein–lipid interactions. Emulsifying stability (ES) referred to the ability of an emulsion to resist creaming, flocculation, and coalescence (64). As shown in Table 5, the EAI and ES of MP reduced in all feeding groups with the post-mortem time prolonged. Protein structural alterations brought on by oxidation, as shown by surface hydrophobicity and FT-IR studies, affected the protein–protein interactions. When adsorbed on the surface of the oil droplets, the cross-linked MP was too big to maintain their flexibility (65). The supplement of selenium yeast or jujube powder to the diet had a very significant effect on the EAI and ES of chicken MP (*P* < 0.01), but the two only have significant interactions on EAI (*P* < 0.05). Compared with the CK group, the EAI and ES of the 0.6S+J histone increased significantly (*P* < 0.05), which were 1.15 m^2^/g and 39.76%. Among the J group, 0.3S group, 0.6S group, and 0.3S+J group, though the EAI and ES gradually increased, there was no significant difference (*P* > 0.05). Zhao et al. (17) have studied that the high-dose GML feed supplementation improved the egg white protein emulsion stability during long-term storage. The higher surface hydrophobicity was indicative of increased protein flexibility, which led to an increase in repulsion between droplets in the emulsion by hydrophobic interactions. Therefore, the addition of selenium yeast or jujube powder could protect the protein from oxidative degradation resulting in the increase of Trp natural fluorescence, leading to the protein–lipid interaction changes. However, different outcomes were found by Zhao et al. (17) that there were no significant differences in EA or ES of fresh egg yolk from laying hens fed docosahexaenoic acid-rich microalgae (*P* > 0.05), which indicates that DHA enrichment has not changed the emulsification properties of egg yolk.

#### Foaming capacity and foaming stability

The foamability depends on the relationship between the final volume of the solution and the volume of the formed foam, while foam stability is based on the ability of the protein to stabilize the foam against gravitational stress (66). Changes in the foaming capacity (FC) and foaming stability (FS) of chicken MP are shown in Table 5. The FC and FS of post-mortem chicken MP of all treatments were reduced with aging time prolonged. As mentioned earlier, the surface hydrophobicity of MP was increased during storage, as Dong et al. (67) indicated that FS is primarily influenced by protein concentration, hydration, and interactions between hydrophobic residues. The addition of selenium yeast and jujube powder in the diet makes the foamability and foam stability of chicken MP, thus showing an upward trend after slaughter. With the increase in selenium yeast addition level, the foaming property and foam stability increased significantly (*P* < 0.05); and compared with the treatment group without jujube powder at the same level, the foaming property and foam stability of the group treated with compound jujube powder were higher. Compared with the CK group, the addition of selenium yeast and jujube powder to the diet can significantly increase the foaming of chicken MP (*P* < 0.05). Studies have shown that the addition of selenium yeast and jujube powder to the diet can increase the foaming and foam stability of chicken myofibril protein, and the combination of the two has the best effect. The 0.6S+J group chicken myofibril protein foams had the highest performance and foam stability. Foaming properties of EWP which were got from dietary GML supplementation have been studied, and the same results were found (17). The increase in FC induced by dietary GML supplementation is associated with the effects of GML on the protein composition and concentration of EWP, which lead to the exposure of more hydrophobic groups and improved protein–air interactions. Consistent with our results, dietary selenium yeast and jujube powder supplementation induced partial unfolding of the protein structure, leading to rapid adsorption at the freshly formed air–water interface and forming viscoelastic films, thus leading to greater stability (66).

### Changes in the gelation properties of myofibrillar protein

Gel strength is an important functional property of heat-induced MP gels, and protein gelation is the most important texture formation property of processed meat products (68). The effects of dietary selenium yeast and jujube powder on the gel properties of MPs in white broilers are shown in Table 6. With an increase in selenium yeast and jujube powder concentration, the strength, springiness, and water-holding capacity of chicken MP heat-induced gel increased. Compared with that in the CK group, the MP gel properties in the 0.6 S + J group increased with aging time. At 0, 24, and 72 h post-mortem, the strength of MP gel was increased by 21.00 g, 17.35 g (*P* < 0.05), and 17.02 g (*P* > 0.05); the gel springiness was increased by 0.04, 0.04, and 0.05% (*P* > 0.05); and the gel WHC was increased by 0.10% (*P* < 0.05), 0.08% (*P* > 0.05), and 0.12% (*P* < 0.05), respectively. However, the effects of selenium yeast and jujube powder on the gelation properties of chicken MPs were not significant, and there was no significant interaction between them. Except in the CK group, there were no significant differences in gel strength and water-holding capacity in the other feeding groups, and gel springiness showed significant differences only between the CK and high-dose compound groups. The results showed that the combination of high-dose selenium yeast and jujube powder could significantly improve the strength, springiness, and water-holding capacity of chicken MP gel after slaughter.

**TABLE 6:** Changes on gel properties of post-mortem chicken myofibrillar protein from selenium yeast and jujube powder dietary supplements.

Items	Time/h	Gel strength/g	Gel springiness/%	Gel WHC/%
CK	0	111.11 ± 12.15^a^	0.95 ± 0.02^c^	0.39 ± 0.07^b^
	24	120.75 ± 14.31^a^	0.94 ± 0.03^ac^	0.39 ± 0.09
	72	121.59 ± 4.42	0.93 ± 0.05^a^	0.35 ± 0.03^c^
CK+J	0	109.35 ± 4.70^a^	0.95 ± 0.03^bc^	0.41 ± 0.06^ab^
	24	114.51 ± 13.78^ab^	0.95 ± 0.03^bc^	0.40 ± 0.04
	72	115.58 ± 16.42	0.95 ± 0.03^ab^	0.39 ± 0.07^bc^
0.3S	0	108.91 ± 11.93^a^	0.97 ± 0.02^abc^	0.42 ± 0.05^ab^
	24	112.49 ± 13.15^ab^	0.96 ± 0.02^abc^	0.42 ± 0.05
	72	113.70 ± 14.42	0.96 ± 0.02^ab^	0.41 ± 0.04^ab^
0.6S	0	105.18 ± 19.25^ab^	0.97 ± 0.02^ab^	0.43 ± 0.06^ab^
	24	108.72 ± 8.34^ab^	0.97 ± 0.02^abc^	0.43 ± 0.05
	72	109.07 ± 14.55	0.97 ± 0.02^a^	0.43 ± 0.04^ab^
0.3S+J	0	102.38 ± 17.52^ab^	0.97 ± 0.02^a^	0.44 ± 0.10^ab^
	24	107.40 ± 12.41^ab^	0.98 ± 0.01^ab^	0.44 ± 0.07
	72	108.59 ± 20.36	0.98 ± 0.01^a^	0.44 ± 0.07^ab^
0.6S+J	0	90.11 ± 12.52^b^	0.99 ± 0.00^a^	0.49 ± 0.06^a^
	24	103.40 ± 10.57^b^	0.98 ± 0.00^a^	0.47 ± 0.06
	72	104.57 ± 16.07	0.98 ± 0.01^a^	0.47 ± 0.03^a^
SEM		13.551	13.551	0.055
*P*	S	<0.01	<0.01	<0.01
	J	<0.05	<0.05	<0.01
	S × J	0.840	0.840	0.689

A: Different lowercase letters represent the same time, and the difference between different treatment groups is significant (P < 0.05); in P-value, S represents the influence of Selenium yeast factor, J represents the influence of Jujube powder, and S × J represents the interaction effect of surface.

Wang et al. (69) found that GluOx-mediated H_2_O_2_ oxidation was much more controlled and could lead to appropriate structural changes in MP, thereby increasing the coordination of protein–protein associates and aggregates and producing firmer and more elastic MP gels. This explains why modifying the protein oxidation status by Se supplementation may change the gel properties. To improve the stability and palatability of muscle meals, Lv et al. (70) investigated the presence of additional inhibitors of MP oxidation using antioxidants such as phenolic compounds. Incorporating phenolic substance (i.e., catechin) into pork MP improved the gelling capability and structural integrity of the gel by potentially involving both intra- and inter-molecular interactions. The active ingredients in jujube powder include phenolic compounds and antioxidants, and molecular interactions may be the reason for the changes in gel properties. According to Balange and Benjakul (71), tannic acid promotes the production of sarcoplasmic protein aggregates, which interfere with the cross-linking of actomyosin and tannic acid, thereby compromising the gel characteristics of mackerel muscle meat.

## Conclusion

Dietary supplementation with selenium yeast and jujube powder significantly affected the conformation and functional characteristics of MPs in white feather broilers. Compared with that in the control group, the increase in the level of selenium yeast in the diet or the addition of jujube powder resulted in a significant increase in the α-helix content of chicken MP (*P* < 0.05) and a significant decrease in the β-sheet content (*P* < 0.05). Furthermore, the endogenous fluorescence intensity increased; solubility, emulsification, and foaming of chicken myofibrils were significantly improved; turbidity was significantly reduced; and the strength, elasticity, and water-holding capacity of the heat-induced gel were also improved in a dose-dependent manner. Therefore, the structural integrity and functional properties of MPs increase with increasing selenium yeast levels. Selenium yeast and jujube powder interact significantly with the secondary structure of MPs to affect the turbidity, emulsification, and foam stability of the protein.

In summary, the addition of selenium yeast and jujube powder to the diet can be used as an important means of pre-slaughter oxidation modification to produce antioxidant effects in animals, delay the body’s post-mortem oxidation reaction speed, and maintain the muscle MP structure post-mortem. Functionality and structure are closely intertwined. Owing to the physicochemical properties of proteins, protein oxidation may cause changes in functional characteristics. Functionality improvement is typically observed in the textural characteristics of comminuted and restructured meat products. Therefore, in the future, feed additives may be a useful approach to improve meat processing properties and enhance the nutritional and health benefits of meat by modifying muscle protein properties. However, it is necessary to conduct additional research to explore the mechanism through which feed additives change muscle properties and to establish the quantity and types of natural antioxidants necessary to significantly improve meat processing properties.

## Data availability statement

The raw data supporting the conclusions of this article will be made available by the authors, without undue reservation.

## Ethics statement

The study was approved by Ethics Committee of Gansu Agricultural University, all methods were carried out in accordance with relevant guidelines and regulations.

## Author contributions

ZW: methodology, investigation, and writing – original draft. CY: software, visualization, and investigation. XY: methodology, resources, and investigation. LZ: conceptualization, funding acquisition, supervision, and writing – review and editing. QY: supervision. DT: project administration. All authors have read and agreed to the published version of the manuscript.
